# High Prevalence of HTLV-1 Infection among Japanese Immigrants in Non-endemic Area of Brazil

**DOI:** 10.1371/journal.pntd.0003691

**Published:** 2015-04-17

**Authors:** Larissa M. Bandeira, Silvia N. O. Uehara, Marcel A. Asato, Gabriela S. Aguena, Cristiane M. Maedo, Nikolas H. Benites, Marco A. M. Puga, Grazielli R. Rezende, Carolina M. Finotti, Gabriela A. Cesar, Tayana S. O. Tanaka, Vivianne O. L. Castro, Koko Otsuki, Ana C. P. Vicente, Carlos E. Fernandes, Ana R. C. Motta-Castro

**Affiliations:** 1 Federal University of Mato Grosso do Sul, Campo Grande, Mato Grosso do Sul, Brazil; 2 Central Public Health Laboratory, LACEN/MS, Camp Grande, Mato Grosso do Sul, Brazil; 3 Laboratory of Molecular Genetics of Microorganisms and Oswaldo Cruz Institute, Rio de Janeiro, Brazil; 4 Oswaldo Cruz Foundation, Campo Grande, Mato Grosso do Sul, Brazil; George Mason University, UNITED STATES

## Abstract

**Background:**

Human T-lymphotropic virus type 1 (HTLV-1) has worldwide distribution and is considered endemic in many world regions, including southwestern Japan and Brazil. Japanese immigrants and their descendants have a high risk of acquiring this infection due to intense population exchange between Brazil and Japan.

**Objective:**

This cross-sectional study aimed to estimate the prevalence of HTLV, analyze the main risk factors associated with this infection, identify the main circulating types and subtypes of HTLV in Japanese immigrants and descendants living in Campo Grande-MS (Middle-West Brazil), as well as analyze the phylogenetic relationship among isolates of HTLV.

**Study Design:**

A total of 219 individuals were interviewed and submitted to blood collection. All collected blood samples were submitted for detection of anti-HTLV-1/2 using the immunoassay ELISA and confirmed by immunoblot method. The proviral DNA of the 14 samples HTLV- 1 positive were genotyped by nucleotide sequencing.

**Results:**

The overall prevalence of HTLV-1 was 6.8% (IC 95%: 3,5-10,2). Descriptive analysis of behavioral risk factors showed statistical association between HTLV-1 and age greater than or equal to 45 years. The proviral DNA of HTLV-1 was detected in all HTLV-1 positive samples. Of these, 14 were sequenced and classified as Cosmopolitan subtype, and 50% (7/14) belonged to subgroup A (transcontinental) and 50% (7/14) to the subgroup B (Japanese).

**Conclusion:**

The high prevalence of HTLV-1 found evidence of the importance of early diagnosis and counseling of individuals infected with HTLV-1 for the control and prevention of the spread of this infection among Japanese immigrants and their descendants in Central Brazil.

## Author Summary

The population of Okinawan immigrants is considered vulnerable to human T-lymphotropic virus type 1 (HTLV-1) infection because the Okinawa region in Japan is an endemic area. The second Brazilian largest Okinawan community is set in Campo Grande, Middle-West Brazil. This study aimed to estimate the prevalence and risk factors associated with HTLV infection among Japanese immigrants and their descendants living in Campo Grande. The prevalence of 6.8% of HTLV-1 infection that was found is considered high. The HTLV-1 infection was associated with age ranged from 45 years old or older. Most infected individuals are Okinawan descendants. The HTLV-1 rate found in the present study indicates that the prevalence of this infection remains high among this Japanese community. This study emphasizes the importance of implementing preventive and diagnostic public health policies to decrease the risk of HTLV-1 transmission among Japanese communities throughout Brazil.

## Introduction

The retrovirus human T-lymphotropic virus type 1 (HTLV-1) is associated with many severe diseases, including adult T-cell leukemia/lymphoma (ATL) and HTLV-1-associated myelopathy/tropical spastic paraparesis (HAM/TSP), but most infected people remain asymptomatic [1]. Seven genetic subtypes have been defined (a-g) based on analyses of the HTLV-1 long terminal repeat (LTR) region. The Cosmopolitan subtype (1a) is the most widespread [2].

Approximately 10 million people are estimated to be infected with HTLV-1 throughout the world [2]. A high prevalence of HTLV-1 infection can be found in the endemic regions of equatorial Africa, the Caribbean islands, Japan, Colombia, northeast Australia, Papua New Guinea and Brazil, that has heterogeneous geographic distribution. However, the highest infection rate has been observed to occur in the islands of Kyushu and Okinawa, in southwestern Japan, and Hokkaido, in north of Japan, with approximately 1.1 million of infected individuals [3,4]. In Brazil, HTLV-1 was first described in 1986 among Japanese immigrants from Okinawa, Southern Japan, residing in the city of Campo Grande, state of Mato Grosso do Sul. Prevalence rates of 13% in the immigrants and 8% in their descendents were observed [5].

Since confirmatory tests for the diagnosis of HTLV infection were not available in the 80s, the prevalence found by Kitagawa and cols (1986) could be overestimated due to the presence of false positives. Therefore, considering the lack of regional studies on HTLV infection, the Japanese immigration wave to Brazil, particularly to Mato Grosso do Sul state and the risk of intrafamilial transmission of HTLV-1, the main goal of this study was to revisit the situation of HTLV-1 epidemiology, especially its estimated prevalence and molecular characterization around 30 years after the first published epidemiological study in Japanese immigrants and their descendants living in Campo Grande, MS.

## Materials and Methods

### Study design

This cross-sectional study was conducted between April 2012 and October 2013 in the city of Campo Grande, capital of the state of Mato Grosso do Sul in midwestern Brazil, which has about 786,797 inhabitants with an estimated contigent of 20,000 Japanese-Brazilian descendants from 4,000 families, showing high migration of this population to Campo Grande [6,7].

In order to get a statistical power of 80% (β = 20%), a significance level of 95%(α <0.05) and an accuracy of 4%, the calculated sample size to estimate the prevalence of HTLV-1 infection, should include at least 216 individuals, based on the prevalence of 10.0% for anti-HTLV-1/2 found by Kitagawa et al. (1986). Two hundred and twenty two potential subjects met the sample criteria. Of them, 219 (88.6%) agreed to participate. Therefore, the study population included 219 participants, who were from, approximately, 70 families in a population of about 18,000 Japanese immigrants and Japanese descendants living in Campo Grande-MS. Individuals were eligible to participate if they were older than 18 years of age, Japanese immigrant (born in Japan), Japanese descendant (had any genetic relationship to a Japanese immigrant) and non-descendant (had relationship to a Japanese immigrant only by marriage) and able to provide written informed consent. Pregnant women were excluded from the study. The presence of other illness or other infections was not used as selection criteria. The participants were previously advised about the campaign through posters and leaflets placed in the Okinawa Association and Brazilian Nipo Association. In addition to anti-HTLV test, free testing for diabetes and blood pressure were offered by the health campaign. The participants did not know their serological profile for anti-HTLV-1/2.

They were informed of the objectives and methods of the study and written informed consent was obtained from all study participants prior to enrollment. Following enrollment, a questionnaire was administered to obtain sociodemographic characteristics and identify risk factors associated with HTLV transmission, including age, gender, immigrant generation, history of residence in Japan, history of blood transfusion, surgery, personal item/sharps sharing, injection drug use, breastfeeding, multiple partners, unprotected sex and sexually transmitted diseases.

Blood samples were collected from study participants and serum samples were evaluated by an enzyme-linked immunosorbent assay (ELISA) test for the presence of anti-HTLV 1 and 2 antibodies (MP Diagnostic HTLV-1/2 ELISA 4.0—MP Biomedicals, Singapore) and confirmed by Western Blot (WB) assay with a commercial test (MP Diagnostics HTLV BLOT 2.4–Singapore). The samples reactive by screening and positive by WB were considered positive for HTLV-1 or 2 infections. DNA was extracted from whole blood samples using the DNA Genomic Purification Kit (Wizard^®^ Genomic, Promega, Madison, WI, USA), according to manufacturer’s instructions. Nested PCR amplified the targeted portion of the 5’LTR of HTLV-I. Reactions were performed as previously described [8]. Internal primers were HFL9 (AAGGCTCTGACGTCTCCCCCC) and HFL10 (TCCCGGACGAGCCCCCAA), corresponding to nucleotide positions 124–144 and 779–796 from the ATK-1 sequence. Products of the second PCR reaction with molecular-weight size marker were subjected to electrophoresis on 1.8% agarose gel in TAE buffer (Tris-acetate EDTA) at 70V for 30 minutes. After being stained with ethidium bromide, gels were visualized on the Kodak Gel Logic 112 photo documentation system. The LTR 672 bp amplicons were purified using illustra^TM^ PCR DNA and Gel Band Purification Kit (GE Healthcare) according to the manufacturer’s instructions. The fragments were sequenced using BigDye Terminator Cycle Sequencing Ready reaction Kit and ABI 1373 (Applied Biosystems, Foster City, CA, USA). Nucleotide sequences were aligned and compared with published HTLV-1 sequences from various geographic regions using ClustalX and Mega 5.05. Philogenetic trees were constructed by use of the neighbor-joining (NJ) method. The NJ tree was evaluated by bootstrap analyses of 1000 replicates.

Data were entered into Epi Info 7.1.2.0 (Centers for Disease Control and Prevention [CDC], Atlanta, GA), a statistical software package. Statistical analyses were performed using SPSS Statistics Data Editor (Statistical Package for Social Science, Chicago, Illinois, USA). HTLV prevalence was estimated using 95% confidence intervals (95% CI). Potential risk factors for HTLV infection were assessed in univariate analysis. Chi-square and Fisher’s exact tests were performed for comparison of categorical parameters. Chi-square and Fisher tests were two-tailed. Statistical significance was considered at p≤0.05.

### Ethics statement

The present study was approved by the Ethics Committee on Research Involving Human Subjects of the Federal University of Mato Grosso do Sul (CEP/UFMS), under protocol number 2249 CAAE 0329.0.0.049.000–11. All adult subjects provided written informed consent, and a parent or guardian of any child participant provided written informed consent on their behalf.

## Results

A total of 219 individuals, Japanese immigrants or descendants and non-descendants of Japanese immigrants living in Campo Grande-MS were enrolled in this study. Most of those were female (61.2%). Mean age was 54.4 years (range 11 to 101 years) and the majority (69.9%) had 45 years of age or older.

The majority (88.1%) was born in Brazil and 10.9% were born in different regions of Japan (Okinawa, Tokyo, Yamaguchi, Hokkaido, Aichi and Kumamoto) and 1.0% was from Japanese descendants from Peru. The Brazilian group (n = 193) was composed of 187 (96.8%) Japanese descendants and 6 (3.1%) non-Japanese descendants. The majority (54.8%) of participants reported having a steady sexual partner (married) or cohabitating with a sex partner. Regarding education, 74% had completed secondary education or college.

The prevalence of HTLV-1 infection was 6.8% (95% IC: 3.5 to 10.2). None of the 219 individuals presented anti-HTLV-2 antibodies. None of the positive subjects showed clinical signs compatible with HAM/TSP or ATL, and therefore, were classified as asymptomatic. The frequency of anti-HTLV seropositivity was higher among Okinawan descendants (8.1% vs. 2.1%) than among descendants from other regions of Japan and non-Japanese descendants; however, this difference was not statistically significant.

Among studied population, a frequency of 6.1% of HTLV-1 infection was found among Japanese-Brazilian descendants participants, while 12.5% was observed in those who declared themselves as foreign, which comprise 22 Japanese and 2 Peruvian nationals. Most infected subjects (n = 14) claimed to be Okinawan descendant, and only one who was not a descendant was positive for anti-HTLV.

**Fig 1:**
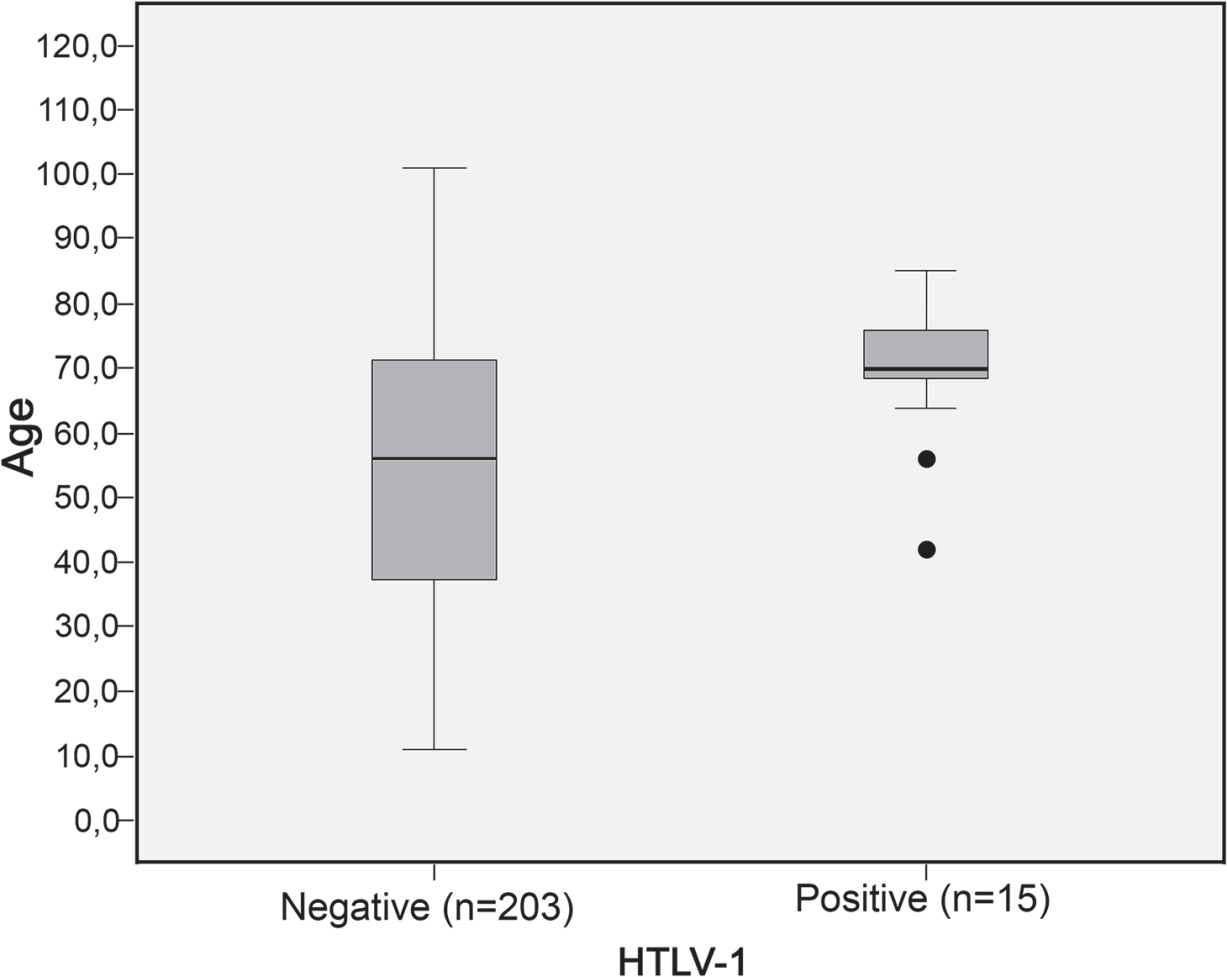
Box plot (interquartile intervals and ranges) for the age distribution (years) of anti-HTLV-1 seropositive and anti-HTLV-1 seronegative individuals from the Japanese community of Campo Grande-MS (n = 219). Whiskers represent maximum 1.5 IQR.

Risk factors for HTLV infection among the studied population are shown in Table 1. In the univariate analysis, HTLV-1 infection was associated only with age ≥ 45 years (*P* = 0.04). The mean age of subjects positive for HTLV-1 was 70 years, which was greater than that of 53 years for non-reactive subjects. Fig 1 shows the variation of age in individuals negative and positive for HTLV-1. Age of infected participants was concentrated in the seventh decade of life, with only two outliers representing younger infected individuals (between 40 and 60 years). However, the age of uninfected people ranged from 11 to 101 years, mainly in the range of 40 to 70 years. All infected subjects were breastfed as children; however, this risk factor was not statistically significant. Some risk factors were absent or infrequently reported in this population, such as history of tattooing, injection drug use and multiple partners.

**Table 1:** Descriptive analysis of risk behaviors among Japanese immigrants and Japanese descendants living in Campo Grande, MS, Brazil, 2012–2013 (n = 219).

Characteristic		HTLV	%	*P*
		Positive/Total		
**Gender**				
	Male	5/85	5.8	
	Female	10/134	7.5	0.65
**Age (years)**				
	<45	1/66	1.5	
	≥45	14/153	9.1	0.04
**Marital status**				
	Married	7/120	5.8	
	Single/divorced/widow	8/99	8.8	0.51
**Birthplace**				
	Brazil	12/195	6.1	
	Other	3/24	12.5	0.24
**Education (years)**				
	>12	5/85	8.4	
	10–12	5/75	6.7	0.83
	≤9	5/59	8.5	0.55
**Descendent of Okinawan**				
	No	1/47	2.1	
	Yes	14/172	8.1	0.15
**History of residence in Japan**				
	No	7/133	5.3	
	Yes	8/86	9.3	0.25
**History of breastfeeding**				
	No	0/3	0.0	
	Yes	15/216	6.9	0.64
**History of blood transfusion**				
	No	13/195	6.7	
	Yes	2/24	8.3	0.76
**Family heritage**				
	Non-Japanese descendent	1/8	12.5	
	Japanese descendent	12/187	6.4	0.50
	Japanese immigrant	2/24	8.3	0.73
**History of surgery**				
	No	3/77	3.9	
	Yes	12/142	8.4	0.20
**Sharing needlestick objects**				
	No	11/149	7.4	
	Yes	4/70	5.7	0.65
**Condom use**				
	Yes	3/55	5.4	
	No	12/142	8.4	0.48
	Does not apply (22)			
**History of STD**				
	No	8/148	5.4	
	Yes	7/71	9.8	0.82
**Sexual partner with a history of blood transfusion**				
	No	12/201	5.9	
	Yes	3/18	16.7	0.08

**Fig 2:**
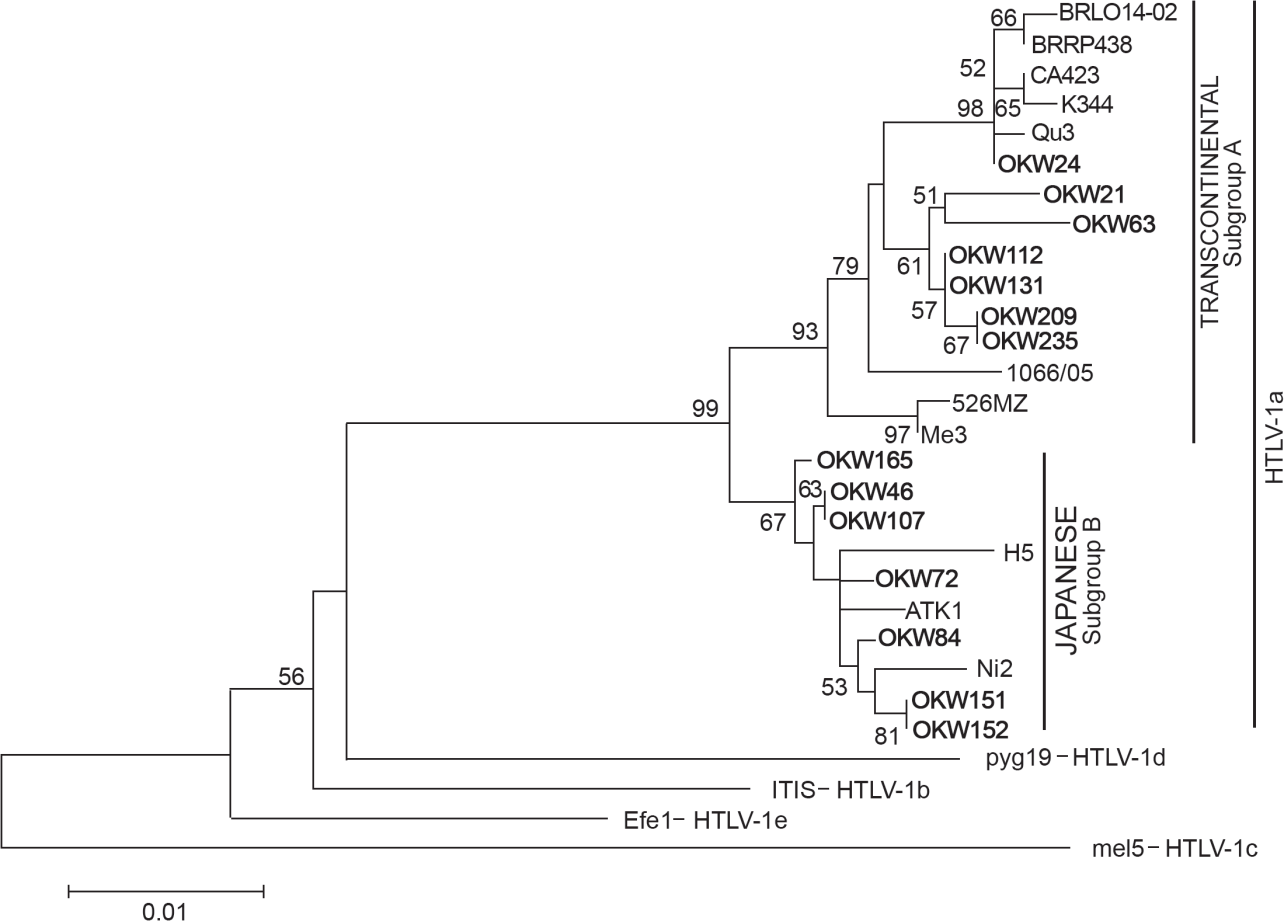
Phylogenetic tree constructed by neighbor-joining (NJ) method using the Kimura-2-parameters distance model for the partial LTR region of 635bp of HTLV-1 showing the phylogenetic relationships among 14 isolates of proviral DNA of HTLV-1 studied (OKW) with 15 isolates available in GenBank.

The proviral DNA of HTLV-1 was detected by amplification of the 5 'LTR region by nested PCR in all samples (n = 15) HTLV-1 positive. Of them, 14 were sequenced and classified as Cosmopolitan subtype and 50% (7/14) belonged to subgroup A (Transcontinental) and 50% (7 / 14) to the subgroup B (Japanese). The new nucleotide sequence samples described in this study have been deposited in GenBank with accession number as follows: OKW21 (KM023750), OKW24 (KM023751), OKW46 (KM023752), OKW63 (KM023753), OKW72 (KM023764), OKW84 (KM023754), OKW107 (KM023761), OKW112 (KM023755), OKW131 (KM023756), OKW151 (KM023757), OKW152 (KM023758), OKW165 (KM023759), OKW209 (KM023760), OKW235(KM023767) were compared with nucleotide sequences of 17 isolates of HTLV-1 available from GenBank (Fig 2). The GenBank accession numbers for the sequences of the 5 'LTR region of HTLV-1 included in the phylogenetic analysis are: ATK1 (J02029), H5 (M37299), pyg19 (L76310), ITIS (Z32527), Me3 (Y16480), CA423 (EU108724), Mel5 (Lo2534), K344 (GQ443755), BRLO14-02 (JF271836), BRRP438 (DQ323811), Qu3 (Y16477), 1066/05 (HQ606137), 526MZ (GU194504), Ni2 (Y16487), efe1 (Y17014). The HTLV-1aA strains of this study clustered closely with other isolates from Latin America, mainly from Brazil. Further, the HTLV-1aB strains of this study clustered closely with other isolates from Japan and from a Japanese descendant from Peru.

## Discussion

Currently, there are approximately an estimated 1.5 million Japanese descendants living in Brazil, whose vast majority reside in the state of São Paulo (Southwest region), followed by the state of Paraná (South region) and Mato Grosso do Sul (Central-West region). In Brazil, 10% of the descendants of Japanese immigrants originate from the province of Okinawa, and the second largest Okinawan community in Brazil is in Mato Grosso do Sul State [9].

The population of Okinawan immigrants is considered at risk of acquiring HTLV-1 due to originating from the endemic islands of Okinawa, Japan, where the prevalence of HTLV-1 varies between 14% and 31% [4].

In the present study, 6.8% HTLV-1 infection prevalence was observed. This percentage is considered high, indicating there should be greater attention given to HTLV-related diseases in this population. This prevalence is 40 and 52 times higher than those found in prime blood donors (0.17%) and pregnant women (0.13%) from Campo Grande (Central-West Brazil), respectively, and 13.6 times higher than that found in African descendants residing in Central Brazil (0.5%) [10,11,12]. In another study conducted with Japanese immigrants in the city of Tome-Açu in northern Brazil, the prevalence (2.8%) was smaller than that found in this study [13].

When comparing the prevalence of HTLV-1 among members of the Japanese community of Campo Grande in the year of 1986 with the current prevalence found in this study, we observed the maintenance of high prevalence over the last 27 years (10% vs 6.8%). This difference was not significant (*P* = 0.28) [5]. Maybe, the prevalence of 6.8% was attributed to the improvement in serological and confirmatory tests over the years [14]. In addition, the prevalence of 10% found in the 1986 study may also be due to the higher number of Japanese immigrants (n = 46) investigated when compared to the current study, in which the majority of subjects were descendants of Japanese immigrants and only 24 were Japanese immigrants. Moreover, all participants in the previous study were descendants of Okinawans, and in the present study, 78.5% of participants had Okinawan ancestry [5].

Increasing age was significantly associated with HTLV-1 infection. This finding was also reported in previous studies and may be due to the cumulative risk of HTLV-1 infection over the lifetime of individuals surveyed [15,16]. The increased prevalence in older individuals may also be a reflection of close kinship to Japanese immigrants from endemic areas, who were likely infected in Japan and brought the virus in the recent past to Brazil. There may have been an age cohort effect resulting from declining HTLV-1 seroprevalence over the past decades. In two studies conducted in Japan, one reported a high seropositivity rate (above 40%) in people over 40 years of age and another, more specifically in Okinawa, found a 21% rate of HTLV-1 in the general population over 40 years [17,18]. Also, a change in the age distribution of patients with HTLV-1 infection in several studies was observed. Given in the 80s most of the patients were between 50–59 years of age, then in 2007, the range would be from 60 to 80 years, as encountered in the present study [19,20]. Although the association between sex and HTLV-1 infection was not statistically significant in our study, higher prevalence of HTLV-1 infection found among females may be due to male-to-female transmission being more efficient during sexual intercourse [21]. These data is in agreement with the prevalence rates in Japan, which show the same pattern of older age and sex-specific prevalence [22].

Although breastfeeding is known to play an important role in the transmission of HTLV-1, it was not statistically significant in our study [23,24]. In 2003, the State Coordination of Epidemiological Surveillance of Mato Grosso do Sul determined the notification of every case of a pregnant woman infected with HTLV as well as provided advice to suspend breastfeeding and started to furnish formula milk. These preventative measures were considered important strategies for controlling the spread of the virus by vertical transmission [11].

All of HTLV-1 isolates were classified as Cosmopolitan subtype (HTLV-1a) and half of them (50%) belonged to subgroup A (Transcontinental) and 50% to subgroup B (Japanese). In Brazil, the Cosmopolitan is the most prevalent subtype of HTLV-1 and the Transcontinental subgroup (HTLV-1aA) is the most prevalent among them, followed by the Japanese subgroup (HTLV-1aB), which is detected among Japanese-descendants and immigrants, such as in the present study [25]. In Japan, the existence of subgroups named Transcontinental and Japanese was reported, so that, the geographical distribution of subgroups presents a difference defined according to ethnic group and origin of HTLV-infected individual. In Okinawa region, most infected individuals with HTLV-1 belongs to the Japanese subgroup of HTLV-1a (65.2%) [26,27]. A study of the population of Japanese immigrants living in Tome-Acu, Para, also found infection with HTLV-1 subgroup Japanese and Transcontinental, the largest part of the Japanese subgroup (HTLV-1aB) [13]. The presence of HTLV-1aB was identified in three voluntary blood donation in São Paulo, asymptomatic carriers, two of whom were descendants of Japanese [28].

All individuals who are positive for HTLV-1 infection in this study were asymptomatic, and therefore, mostly likely unaware of their serological profile. Thus, the individual may remain asymptomatic and continue to infect sexual partners and family [29]. Considering the results here, we emphasize the importance of implementing preventive and diagnostic public health policies to decrease the risk of HTLV transmission among family members of Japanese communities throughout Brazil.

## Conclusion

The HTLV-1 prevalence of 6.8% (95% CI: 3.5 to 10.2) found in the present study indicates the maintenance of high prevalence of this infection in the Japanese community of Campo Grande-MS over the past 27 years. These findings also showed that the prevalence of HTLV-1 infection is distinct among regions of Brazil, and although high, this rate did not contribute to an increase in HTLV-1 infection prevalence among the general Brazilian population. Further HTLV-1 nucleotide sequencing may provide more information on the molecular epidemiology of this infection in Japanese descendants population living in Brazil, which may be helpful in understanding the HTLV-1 transmission in these isolated communities.

## Supporting Information

S1 Checklist STROBE Checklist. (PDF)

## References

[1] Hedayati-Moghaddam MR (2013) A systematic review for estimation of HTLV-I infection in the blood donors of Iran. Iran J Basic Med Sci 16(3):196–201. 24470861 PMC3881243

[2] Gessain A, Cassar O (2012) Epidemiological aspects and world distribution of HTLV-1 infection. Front Microbiol 3(388):1–23. 23162541 10.3389/fmicb.2012.00388 PMC3498738

[3] Matsubara F, Kato Y, Harada K, Koizumi A, Haraguchi K (2014) Detection of antibodies to Human T-cell Leukemia Virus types 1 and 2 breast milk from East Asian Women. Biol Pharm Bull 37(2):311–314. 24492727 10.1248/bpb.13-00558

[4] Yamashita M, Ishida T, Ohkura S, Miura T, Hayami M (2001) Phylogenetic characterization of a new HTLV type 1 from the Ainu in Japan. AIDS Res Hum Retroviruses 17(8):783–787. 11429119 10.1089/088922201750237068

[5] Kitagawa T, Tadokoro H, Fujishita M, Taguchi H, Miyoshi I (1986) Antibodies to HTLV-1 in Japanese immigrants in Brazil. JAMA 256(17):2342 2877100

[6] IBGE—Brazilian Institute of Geography and Statistics. Demographic census 2010. Available in: <http://www.ibge.gov.br> Access in: 20 Feb. 2014.

[7] AYUMI: A Saga da Colônia Japonesa em Campo Grande SABER, Sampaio Barros Editora, 2005.

[8] Martins RM, do Nascimento LB, Carneiro MA, Teles SA, Lopes CL, et al (2010) HTLV-1 intrafamilial transmission through three generations in an isolated Afro-Brazilian community. J Clin Virol 48(2):155–157. 10.1016/j.jcv.2010.03.004 20395170

[9] Jornal MS SHIMBUM. Projeto Dekassegui é modelo de trabalho. Campo Grande, MS. May/2005; 1(6):5.

[10] Freitas GMB. Estudo clínico e epidemiológico da infecção pelos Vírus linfotrópicos de células-T humanas (HTLV-I/II) em doadores de sangue em Campo Grande-MS (1994–2001). (Doctoral dissertation, Dissertação de mestrado, Fundação Oswalddo Cruz, Rio de Janeiro, 2002).

[11] Dal Frabbro MMFJ, Cunha RV, Bóia MN, Portela P, Botelho CA, et al (2008) HTLV 1/2 infection: prenatal performance as a disease control strategy in State of Mato Grosso do Sul. Rev Soc Bras Med Trop 41(2):148–151. 18545834 10.1590/s0037-86822008000200003

[12] Do Nascimento LB, Santos Carneiro MA, Teles SA, Motta-Castro ARC, Otsuki K, et al (2009) Prevalence of infection due to HTLV-1 in remnant quilombos in Central Brazil. Rev Soc Bras Med Trop 42(6):657–660. 20209350 10.1590/s0037-86822009000600009

[13] Vallinoto ACR, Muto NA, Pontes GS, Machado LF, Azevedo VN, et al (2004) Serological and molecular evidence of HTLV-I infection among Japanese immigrants living in the Amazon region in Brazil. Jpn J Infect Dis 57(4):156–159. 15329447

[14] Jacob F, Santos-Fortuna E, Caterino-de-Araujo A (2008) Algorithm of HTLV-1/2 serological screening tests employed by Instituto Adolfo Lutz. Bol Epidem Paul 5(49):12–18.

[15] Blattner WA, Nomura A, Clark JW, Ho GY, Nakao Y, et al (1986) Modes of transmission and evidence for viral latency from studies of human T-cell lymphotropic virus I in Japanese migrant populations in Hawaii. Proc Natl Acad Sci USA 83(13):4895–4898. 3014518 10.1073/pnas.83.13.4895 PMC323850

[16] Murphy EL, Figueroa JP, Gibbs WN, Holding-Cobham M, Cranston B, et al (1991) Human T-lymphotropic virus type I (HTLV-I) seroprevalence in Jamaica. I. Demographic determinants. Am J Epidemiol 133(11):1114–1124. 2035515 10.1093/oxfordjournals.aje.a115824

[17] Kohakura M, Nakada K, Yonahara M, Komoda H, Imai J, et al (1986) Seroepidemiology of the human retrovírus (HTLV/ATLV) in Okinawa where adult T-cell leucemia is highly endemic. Jpn J Cancer Res 77(1):21–23. 2870044

[18] Tajima K, Kamura S, Ito S, Ito M, Nagatomo M, et al (1987) Epidemiological features of HTLV-I carriers and incidence of ATL in na ATL-endemic island: A report of the community-based cooperative study in Tsushima, Japan. Int J Cancer 40(6)741–746. 2891624 10.1002/ijc.2910400605

[19] Watanabe T (2011) Current status of HTLV-1 infection. Int J Hematol 94(5):430–434. 10.1007/s12185-011-0934-4 21969187

[20] Pinto MT, Rodrigues ES, Malta TM, Azevedo R, Takayanagui OM, et al (2012) HTLV-1/2 seroprevalence and coinfection rate in brazilian first-time blood donors: an 11-years follow-up. Rev Inst Med Trop Sao Paulo 54(3):123–129. 22634882 10.1590/s0036-46652012000300002

[21] Eshima N, Iwata O, Iwata S, Tabata M, Higuchi Y, et al (2009) Age and gender specific prevalence of HTLV-1. J Clin Virol 45(2):135–138. 10.1016/j.jcv.2009.03.012 19386541

[22] Nakashima K, Ikematsu H, Hayashi J, Kishihara Y, Mitsutake A, et al (1995) Intrafamilial transmission of hepatitis C virus among the population of an endemic area of Japan. JAMA 274(18):1459–1461. 7474193

[23] Proietti FA, Carneiro-Proietti ABF, Catalan-Soares BC, Murphy EL (2005) Global epidemiology of HTLV-I infection and associated diseases. Oncogene 24(39):6058–6068. 16155612 10.1038/sj.onc.1208968

[24] Pique C, Jones KS (2012) Pathways of cell-cell transmission of HTLV-1. Front Microbiol 3(378):1–14. 23109932 10.3389/fmicb.2012.00378 PMC3479854

[25] Vicente ACP, Otsuki K, Silva E, Iñiguez A (2005) Molecular epidemiology of HTLV-1 and HTLV-2 in brazilian urban areas. In: VIII INTERNATIONAL SYMPOSIUM ABOUT HTLV IN BRAZIL, São Paulo. The Brazilian journal of infections diseases 9(1).

[26] Otani M, Honda N, Xia PC, Eguchi K, Ichikawa T, et al (2012) Distribution of two subgroups of human T-lymphotropic virus type 1 (HTLV-1) in endemic Japan. Tropical medicine and health 40(2):55–8. 10.2149/tmh.2012-02 23097620 PMC3475314

[27] Yamashita M, Ishida T, Ohkura S, Miura T, Hayami M (2001) Phylogenetic characterization of a new HTLV type 1 from the Ainu in Japan. AIDS Res Hum retroviruses 17(8):783–7. 11429119 10.1089/088922201750237068

[28] Segurado AA, Biasutti C, Zeigler R, Rodrigues C, Damas CD, et al (2002) Identification of human T-lymphotropic vírus type I (HTLV-I) subtypes using restricted fragment length polymorphism in a cohort of asymptomatic carriers and patients with HTLV-I associated myelopathy/tropical spastic paraparesis from São Paulo, Brazil. Memórias do Instituto Oswaldo Cruz 97(3):329–33. 12048560 10.1590/s0074-02762002000300009

[29] Zihlmann KF, De Alvarenga AT, Casseb J (2012) Living invisible: HTLV-1-infected persons and the lack of care in public health. PLOS Negl Trop Dis 6(6):770–773. 10.1371/journal.pntd.0001705 PMC3373594 22720112

